# H19-Promoter-Targeted Therapy Combined with Gemcitabine in the Treatment of Pancreatic Cancer

**DOI:** 10.5402/2012/351750

**Published:** 2012-06-03

**Authors:** Vladimir Sorin, Patricia Ohana, Jennifer Gallula, Tatiana Birman, Imad Matouk, Ayala Hubert, Michal Gilon, Avraham Hochberg, Abraham Czerniak

**Affiliations:** ^1^Department of Biological Chemistry, Institute of Life Sciences, The Hebrew University of Jerusalem, Jerusalem 91904, Israel; ^2^Department of Oncology, Hadassah Medical Center, University Hospital, Jerusalem 91915, Israel; ^3^Department of HPB Surgery “A”, Sheba Medical Center, Tel Hashomer, Israel

## Abstract

Pancreatic cancer is the eighth cancer leading cause of cancer-related death in the world and has a 5-year survival rate of 1–4% only. Gemcitabine is a first line agent for advanced pancreatic therapy; however, its efficacy is limited by its poor intracellular metabolism and chemoresistance. Studies have been conducted in an effort to improve gemcitabine treatment results by adding other chemotherapeutic agents, but none of them showed any significant advantage over gemcitabine monotherapy. We found that 85% of human pancreatic tumors analyzed by in situ hybridization analyses showed moderated to strong expression of the H19 gene. We designed a preclinical study combining gemcitabine treatment and a DNA-based therapy for pancreatic cancer using a non viral vector BC-819 (also known as DTA-H19), expressing the diphtheria toxin A chain under the control of the H19 gene regulatory sequences. The experiments conducted either in an orthotopic and heterotopic pancreatic carcinoma animal model showed better antitumor activity following the sequential administration of the vector BC-819 and gemcitabine as compared to the effect of each of them alone. The results presented in the current study indicate that treatment with BC-819 in combination with gemcitabine might be a viable new therapeutic option for patients with advanced pancreatic cancer.

## 1. Introduction

In spite of the significant progress in treatment of different kinds of cancers, pancreatic cancer still has a very low rate of 5-year survival. The majority of patients with carcinoma of pancreas have already unresectable tumor and metastatic disease at presentation. A number of studies have been conducted in an effort to improve actual treatments by combining chemotherapeutic agents and radiotherapy, but none has yet shown more than a 10 month survival result [1, 2]. Given those facts, the need for novel and effective therapies is essential.

Our group previously reported the use of a new DNA-based therapy for cancer treatment in which the plasmid BC-819 (also known as DTA-H19) drives the expression of diphtheria toxin A chain by the regulatory sequence of the H19 gene only into cancer cells [3, 4]. This therapy demonstrated good results in treatment of bladder, colon liver metastases, pancreatic and ovarian cancers [4–8]. We have shown that BC-819 injected directly into the pancreatic tumor induced a cytotoxic effect in cancer cells without affecting the surrounding normal cells. Treatment with BC-819 presents the possibilities of limiting or reversing tumor progression and even shrinking the tumor to a resectable size [7]. The level of DTA transcript monitored by RT-PCR analyses showed the presence of DTA transcript verifying that the intratumoral injection of the plasmid leads to transfection and expression of the DTA gene [7]. Intratumoral injections of BC-819 in a syngeneic hamster orthotopic tumor model or in subcutaneous human pancreatic tumor developed in a nude mouse model showed significant efficacy in decreasing tumor growth progression. However, it is only local therapy which may not have systemic effect. Therefore, this local control therapy should be combined with systemic chemotherapy that, probably, may improve tumor response and survival. The present work evaluates the possible advantages of local treatment of pancreatic cancer with BC-819 in combination with systemic administration of gemcitabine, the standard of care in pancreatic cancer therapy. As these two drugs induce cell growth arrest by two different mode of action, it is hypothesized that an additive effect will be observed which will force the cells to enter into apoptosis.

This work was designed to demonstrate that a combination of effective local control of the tumor with systemic therapy can improve the results of the treatment.

## 2. Materials and Methods

### 2.1. Cell Lines and Drug Solutions

The human ductal adenocarcinoma cell line CRL-1469 was obtained from the American Type Culture Collection (ATCC; Rockville, MD, USA) and cultured in 90% DMEM-F12 medium and 10% fetal bovine serum (FBS). The hamster pancreatic ductal carcinoma cell line PC.1-0 was kindly provided by Dr. Buscail L. (Institut National de la Santé et de la Recherche Médicale U531, Institut Louis Bugnard, Institut Federatif de Recherche-31, Centre Hospitalier Universitaire Rangueil, Toulouse, France) and was cultured in 90% RPMI-1640 medium supplemented with 10% FBS. Antibiotic solution penicillin (180 units/mL), streptomycin (100 *μ*g/mL), and amphotericin-B (0.2 *μ*g/mL) were added to all medium solution.

Gemcitabine (Eli Lilly) was diluted in saline.

### 2.2. Plasmids Preparation

The reporter plasmid Luc-H19 (which expresses the luciferase gene under the control of the H19 promoter) and the BC-819 construct were prepared as previously described [3, 4].

### 2.3. Orthotopic Model for Pancreatic Cancer

The hamster model of pancreatic cancer was described in previous studies [7, 9], 5-6-week-old male Syrian golden hamsters (70–80 grs) were purchased from Harlan and used to generate the orthotopic model. After anesthetization by subcutaneous injection of Ketamine and Xylazine (85 mg/kg and 15 mg/kg, resp.) the pancreas was surgically exposed and hamster pancreatic carcinoma cells (PC1-0) derived from a pancreatic ductal carcinoma induced by N-nitrosobis (2-oxopropyl) amine were injected into the pancreas of 6-7-week old Syrian Golden Hamsters (*n* = 24). After 7 days, 100% of the animals presented a single tumor in their pancreas. The animals were randomly divided into one of the following groups: untreated control, gemcitabine alone, and BC-819 + gemcitabine; each group contained 8 animals. The control and the gemcitabine groups received 25 *μ*L PBS by intratumoral injection, while the BC-819-treated group received 50 *μ*g of BC-819 in a final volume of 25 *μ*L PBS. The intratumoral injection was performed at day 7 following the injection of tumor cells. Gemcitabine (50 mg/kg) treatment was begun from day 11 and given twice weekly by intraperitoneal injection (IP). Tumor diameter measurements were taken before intratumoral treatment and before sacrifice.

Animals were then sacrificed at day 18 at which time the tumors were measured and the abdominal cavity was searched for visible metastases. Tumors were excised, weighed, and their *ex vivo* dimensions were recorded.

The tumor volume was calculated as *V* = *W* ^2^ × *L*/2, where *L* = length and *W* = width, using width as the smaller dimension. Tumor growth progression (TGP) was calculated according to the following formula: TGP  (%) = [(*T* × 100/*t*)] − 100, where *t* is the volume of tumor at beginning of the treatment and *T* is volume of the same tumor at the end of the treatment.

The tumors were fixed in formalin, processed, and embedded in paraffin for pathology studies. All the tumors were histologically defined as pancreatic cancer.

### 2.4. Toxicity Test

The blood of 4 hamsters at each group was collected for the study of renal and liver function, blood counting (including CBC + differential, creatinine, calcium, phosphorus, urea, glucose, bilirubin, total protein, albumin, globulin, cholesterol, alkaline phosphatase, SGPT, SGOT, sodium, potassium, chloride).

### 2.5. Heterotopic Model for Pancreatic Cancer

*In vivo* local tumor growth experiments were carried out in a subcutaneous xenograft model as described in a previous study [7].

Confluent CRL-1469 human pancreatic carcinoma cells were injected subcutaneously into the back of athymic nude mice (5-6-week-old and 20–30 grams) purchased from Harlan (Zeist, The Netherlands). Tumor-bearing mice were randomized when tumors reached approximately 6 mm diameter. After tumor development (30 days), three BC-819 administrations were performed, with a 2-day interval between each treatment, by direct injection into the tumor at days 0, 2, and 4. Treatment consisted of 25 *μ*g plasmid (BC-819 for treated group and 5% w/v glucose for control group) mixed with the transfectant polyethylenimine (Polyplus, Illkirsh, France) (*N*/*P* ratio = 6) diluted in 50 *μ*L of 5% w/v glucose. Mice were randomly divided into one of the following groups (*N* = 7 per group): control (glucose 5%), BC-819 alone, gemcitabine alone, and BC-819 + gemcitabine. Animals received 2 injections of either gemcitabine (150 mg/kg) or saline by IP administration at days 37 and 41 following cells implantation.

To test survival, an additional xenograft model was used using pancreatic carcinoma cells from hamster [10]. Confluent PC1-0 cells were trypsinized and resuspended in PBS *X* 1. 1.5 × 10^5^ cells in a final volume of 100 *μ*L and were injected subcutaneously into the back of athymic nude mice (5-6-week-old and 20–30 grams). After tumor development (10 days), 3 treatments of plasmid vectors were given, with a 2-day interval between each treatment, by direct injection into the tumor. Treatment consisted of 25 *μ*g plasmid (BC-819 for treated group and Luc-H19 reporter plasmid for control group) mixed with the transfectant polyethylenimine (Polyplus) (*N*/*P* = 6) diluted in 50 *μ*L of 5% w/v glucose. Mice were randomly divided into 4 groups (*N* = 4), including Luc-H19 (control), BC-819, Luc-H19 + gemcitabine, BC-819 + gemcitabine. Animals received either gemcitabine (150 mg/kg) or saline by IP administration at days 17 and 20 following cells implantation. Mice were sacrificed when tumor reached a diameter larger than 13 mm.

All experiments were performed according to the rules of the Animal Ethics Committee.

## 3. Results

### 3.1. BC-819 Treatment in Two Different Animal Models of Pancreatic Cancer

#### 3.1.1. Antitumor Effect following Sequential Administration of BC-819 and Gemcitabine in a Hamster Orthotopic Pancreatic Carcinoma Model

**Figure 1:**
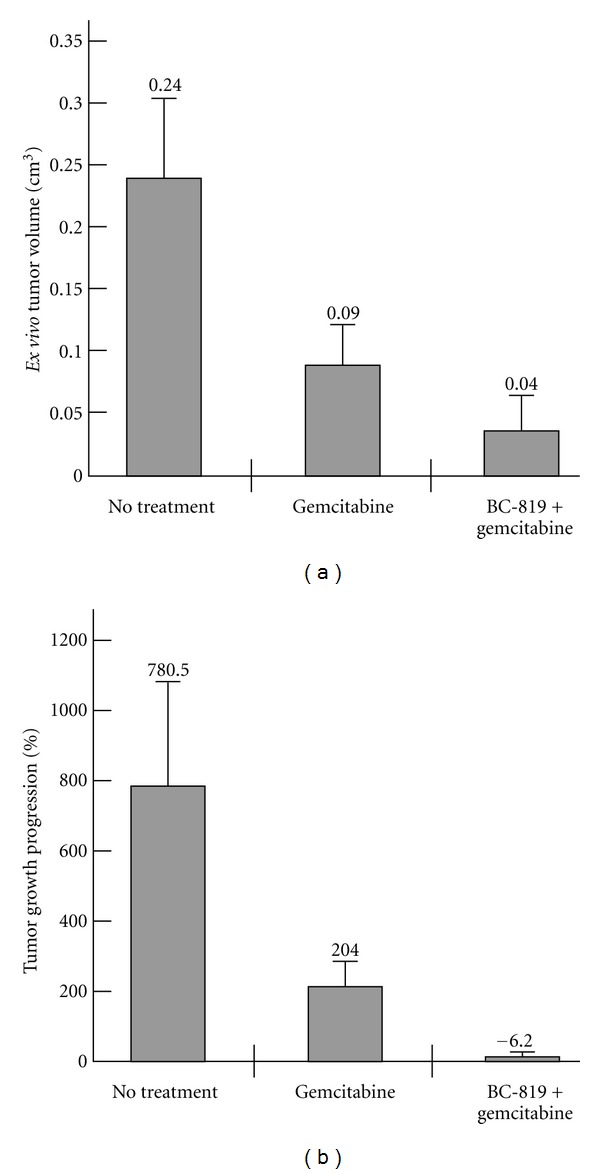
Sequential administration of BC-819 and gemcitabine in a hamster orthotopic pancreatic carcinoma model. (a) Average of *ex vivo* tumor volume of the nontreated, gemcitabine, and BC-819 + gemcitabine treated groups measured after sacrifice (day 18). Asterisks indicate significant difference after BC-819 + gemcitabine compared with no treatment or gemcitabine alone (*P < 0.05, **P < 0.01, and ***P < 0.001). (b) Average of tumor growth progression of the nontreated, gemcitabine, and BC-819 + gemcitabine treated groups calculated as the ratio of tumor size at the end of the experiment (day 18) compared to the size before treatment (day 7).

As expected, gemcitabine treatment was effective in inhibiting the tumor growth as compared to the control group. However, the sequential use of BC-819 and gemcitabine demonstrated further advantages in reducing the tumor burden (Figures 1(a) and 1(b)). The *ex vivo* tumor volume determined at the end of the experiment showed that compared to the control group (no treatment), the gemcitabine group was reduced by 62% while the BC-819 plus gemcitabine group displayed tumors with a size reduced by 83% (*P* < 0.000005). The tumor volume in the sequential use of BC-819 and gemcitabine group was also significantly lower when compared to the gemcitabine group alone (*P* < 0.04) (Figure 1(a)). Similar results were obtained measuring the tumor progression growth (TPG %) (Figure 1(b)). The control group showed 780% increment in tumor size, the gemcitabine group showed approximately 200%, while the BC-819 + gemcitabine group showed a 6% decrease in the tumor growth that is significantly lower than the gemcitabine group alone (*P* < 0.01).

Metastases occurrence was also analyzed: all animals in the control group showed multiple visible metastases at the end of the experiment, as compared to 63% of the animals treated with gemcitabine showing less than 4 metastases, and to 100% of the animals treated with BC-819 and gemcitabine showing no visible metastases (Table 1).

There was no evidence of toxicity in any treatment group based on animal appearance or weight loss. There were also no significant changes in hematology and chemistry in the BC-819 + gemcitabine treated group as compared to the untreated group.

In order to test the systemic toxicity of BC-819 given in combination with gemcitabine, several organ samples were collected from animals treated with BC-819 and gemcitabine at terminal histopathology examination. No gross or microscopic significant alterations were noted in the following organs: pancreas, liver, lung, bowel, spleen, heart, kidney, adrenal gland, lymph node, and gall bladder.

**Figure 2:**
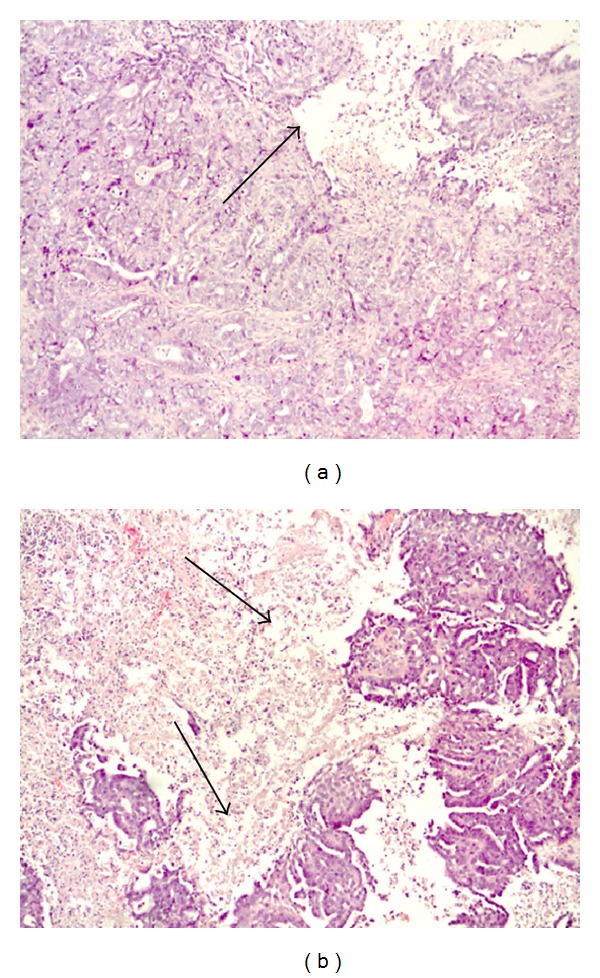
Histopathology analyses in orthotopically induced tumors in the pancreas of hamsters treated either with Luc-H19 + gemcitabine (a) or BC-819 + gemcitabine. (b) The arrows in pictures (a) and (b) mark the necrotic area (X10 original magnification for picture). An extensive area of necrotic tissue it is clearly shown in the BC-819-gemcitabine-treated tumor only. Slides were prepared from paraffin block sections and stained with hematoxylin eosin.

Histopathologic analysis showed that the sequential treatment with BC-819 and gemcitabine increased the necrotic area in the tumor in comparison with control groups (Figure 2).

This study demonstrated that BC-819 used in sequential use with gemcitabine is more efficient than gemcitabine alone at controlling the tumor growth progression (reflected by lower tumors volume) and at preventing the occurrence of metastases.

#### 3.1.2. Antitumor Effect following Sequential Administration of BC-819 and Gemcitabine in a Subcutaneous Human Pancreatic Tumor in Nude Mouse Model

Local human pancreatic tumor growth experiments were carried out in a subcutaneous xenograft model. This animal model has proven useful in the measurement of tumor progression and has been reliable and reproducible.

**Figure 3:**
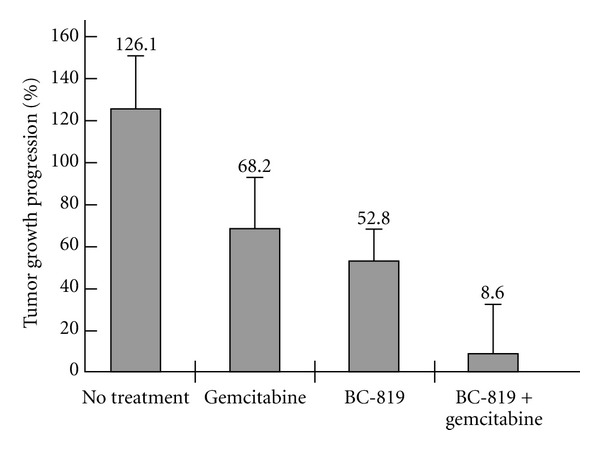
Sequential administration of BC-819 and gemcitabine in a subcutaneous human pancreatic tumor in nude mouse model. and Confluent CRL-1469 human pancreatic carcinoma cells were injected subcutaneously into the back of athymic nude mice. After tumor development (30 days), three BC-819 administrations were performed, with a 2-day interval between each treatment, by direct injection into the tumor at days 0, 2, and 4. Mice were randomly divided into one of the following groups: control (glucose 5%), BC-819 alone, gemcitabine alone and BC-819 + gemcitabine. Average tumor growth progression of control (*N* = 7), BC-819 (*N* = 7), gemcitabine (*N* = 7), and BC-819 + gemcitabine (*N* = 7) groups comparing the tumor volume measured at the end of the experiment to the volume measured at the beginning.

Sequential use of BC-819 and gemcitabine resulted in a significant delay in tumor growth progression as compared to the other groups (Figure 3). The heterotopic model using CRL-1469 cells showed that 3 intratumoral injections of BC-819 alone at 2-day interval or 2 intraperitoneal administrations at 4-day interval of gemcitabine alone were able to decrease tumor growth as compared to the untreated control group; however, the combination of BC-819 and gemcitabine was significantly more effective.

A significant difference in tumor growth progression was found between the group of BC-819 + gemcitabine versus gemcitabine alone (*P* < 0.044) and BC-819 alone (*P* < 0.05) (Figure 3).

Both groups treated with gemcitabine alone or sequentially with BC-819 showed similar weight loss (up to 20%) as compared to the control group or the group treated with BC-819 alone (data not shown).

#### 3.1.3. Evaluation of Survival after Sequential Administration of BC-819 and Gemcitabine in a Subcutaneous Hamster Pancreatic Tumor in Nude Mouse Model

Local tumor growth and survival experiments were carried out in a subcutaneous xenograft model.

**Figure 4:**
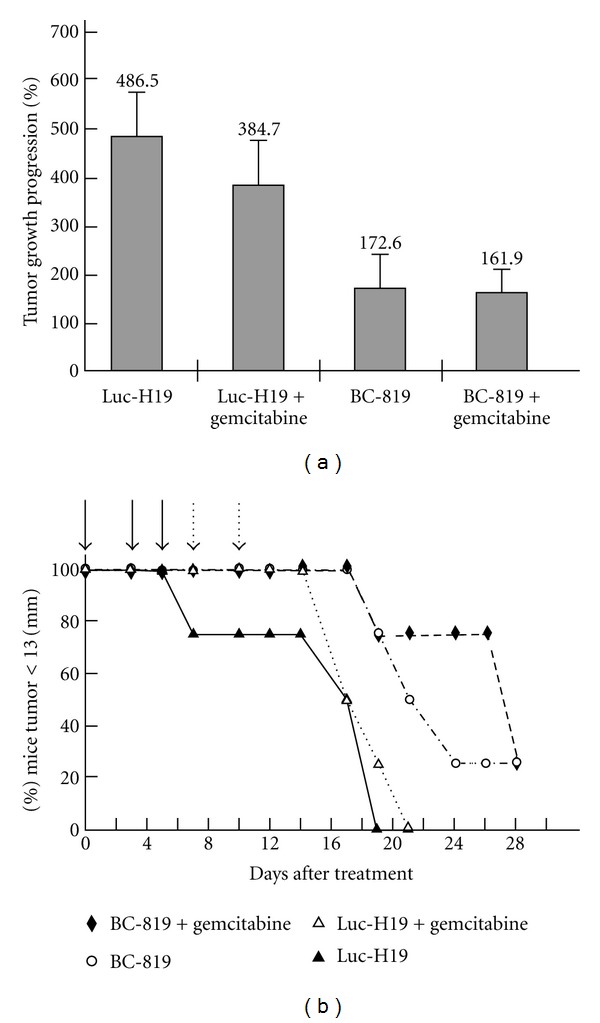
The effect of sequential administration of BC-819 and gemcitabine on tumor progression and on the survival of a nude mice heterotopic pancreatic carcinoma model. Pancreatic carcinoma cells from hamster (PC1-0 cells) were injected subcutaneously into the back of athymic nude mice. After tumor development, 3 treatments of plasmid vectors were given, with a 2-day interval between each treatment, by direct injection into the tumor. Mice were randomly divided into 4 groups of 4 animals each and received the following treatments: Luc-H19 (control vector), BC-819, Luc-H19 + gemcitabine, BC-819 + gemcitabine. Mice were sacrificed when tumor reached a diameter larger than 13 mm. (a) Average tumor growth progression comparing the tumor volume measured after the last treatment to the volume measured before the first treatment. (b) Percentage of mice with a tumor diameter <13 mm as a function of time after the start of the treatment. The days or treatment are marked by arrows.

Sequential use of BC-819 and gemcitabine resulted in a significant difference in tumor growth progression as compared to the other treatment groups (Figure 4(a)). The heterotopic model using PC-1.0 hamster pancreatic carcinoma cells also showed delay in tumor growth after administration of either BC-819 alone or Luc-H19 (control vector) + gemcitabine and further delay when the combination of BC-819 and gemcitabine was used. The inhibiting effect following the combined treatment of BC-819 and gemcitabine in the tumor growth as compared to each of the drugs alone was also more significant in the human pancreatic carcinoma xenograft.

 21 days after the first treatment the survival rate in the group of mice treated with both BC-819 and gemcitabine was 100% while in the group treated with BC-819 alone was 50% and 0% in the groups treated with either the combination of gemcitabine and Luc-H19 or Luc-H19 alone (Figure 4(b)).

A significant difference in tumor growth progression was found between the group of BC-819 + gemcitabine versus Luc-H19 + gemcitabine (*P* < 0.037) (Figure 4(a)).

The results show a significant increase in survival of the group treated with BC-819 + gemcitabine versus Luc-H19 + gemcitabine or BC-819 alone (Figure 4(b)).

Along with moderate differences in the tumor progression between the mice treated with BC-819 alone and those treated with the combination of BC-819 and gemcitabine, survival in the last group was also significantly longer.

## 4. Discussion

The present study proposes a successful approach for the treatment of pancreatic cancer combining conventional chemotherapy used nowadays and a DNA-based therapy expressing a toxin under the control of regulatory sequences of a differentially expressed gene, the H19 gene. The human H19 gene is an imprinted gene, expressed from the maternal allele in several tissues during embryo development and repressed right after birth [11]. H19 gene was shown to be reexpressed in several cancer tissues derived from those tissues which expressed the gene during embryonic development [12] such as bladder cancer [13], hepatocellular carcinoma [14], adrenocortical tumors [15], choriocarcinoma [16], colorectal cancer [17], ovarian carcinoma and lung carcinoma [18]. H19 RNA shares all known characteristics of messenger RNA but has no known protein product [19]. H19 RNA contributes significantly to several aspects of the malignant phenotype including proliferation, hypoxic stress response, angiogenesis, metastasis, and multidrug resistance [20, 21]. Cancer cells devoid of H19 expression encounter a very significant retardation of tumor growth *in vivo* [22]. It was previously demonstrated that the BC-819 construct was able to kill tumor cells both *in vitro* and *in vivo* in animal models for bladder cancer, ovarian cancer, colorectal liver metastases [3–6, 8]. An expression of H19 gene in 85% of the analyzed human pancreatic cancer samples determined by *in situ* hybridization analysis allowed us to suggest effectiveness of the BC-819 construct in this disease. Indeed, direct intratumoral injections of BC-819 caused tumor growth retardation in pancreatic cancer animal models [7].

However, this treatment delaying tumor growth may not efficiently prevent metastatic spread. The combination of local therapy with systemic chemotherapeutic agent is very likely to be more effective in the control of the disease [23–26]. Thus, intraperitoneal administration of gemcitabine may inhibit metastatic spread simultaneously reducing primary tumor growth. Both BC-819 and gemcitabine display a pharmacological effect on cellular growth inhibition gemcitabine acts upstream in the cell cycle by inhibiting DNA synthesis in all cells engaged in mitosis [23–26] while BC-819 acts downstream by inhibiting protein synthesis in tumor cells exhibiting high H19 gene expression levels [3]. As these two products induce cell growth arrest by two different mode of action, it is hypothesized that an additive effect will be observed which will force the cells to enter into apoptosis. Indeed, in both heterotopic and orthotopic pancreatic cancer animal models, intraperitoneal gemcitabine treatment affected tumor growth and reduced the number of developed metastases as compared to the control group. Nevertheless, as we suggested, the sequential use of BC-819 and gemcitabine was found significantly more effective. It provided not only delay in tumor progression, but regression of tumor size in contrast to other groups (Figures 1(a) and 1(b)). The *ex vivo* tumor volume in the sequential use of BC-819 and gemcitabine group was significantly lower when compared to the gemcitabine group alone (*P* < 0.04) (Figure 1(a)). Our previous studies [7] showed that BC-819 treatment was effective in inhibiting the tumor growth compared with the Luc-H19 treated tumors; thus, excluding a nonspecific effect of the BC-819 plasmid and supporting an specific antitumor effect. The orthotopic experiment also showed that the combination of BC-819 and gemcitabine treatment affects metastasis occurrence stronger than gemcitabine alone. In contrast to the control group where all the animals showed multiple visible metastases at the end of the experiment, 63% of the animals treated with gemcitabine alone showed a few number of metastases, whereas 100% of the animals treated with BC-819 and gemcitabine did not show visible metastases at all (Table 1). Tumor invasion and metastasis are the major causes of treatment failure and death in pancreatic cancer. Therefore, the prevention of metastatic spread in animals treated with the sequential administration of BC-819 and gemcitabine indicates that the combined treatment may overcome the limitations associated with single molecule treatment that may also explain the benefit in survival rate in the heterotopic model treated with sequential administration of BC-819 and gemcitabine.

To date, some gemcitabine-containing combinations have demonstrated modest improvement when compared to gemcitabine alone, particularly in patients with good performance status [27, 28]. Delay of tumor progression, decrease in the metastatic process, and increase in the survival rate were achieved combining the systemic effect of gemcitabine with local destruction of tumor by BC-819. These findings demonstrate potential advantages of combination therapy strategies targeting multiple pathways in pancreatic cancer treatment. The rationale to use BC-819 in combination with gemcitabine in patients with locally advanced adenocarcinoma of the pancreas is thus to maximize tumor cell death. BC-819 given locally in combination with systemic chemotherapy may provide additional therapeutic benefit for the treatment of pancreatic cancer.

## Figures and Tables

**Table 1:** Metastases observation developed in the hamster orthotopic pancreatic carcinoma model. Abdominal cavity was searched for metastases in the animals groups described in Figures 1(a) and 1(b).

Treatment	No metastases	Few metastases (<4)	Numerous metastases (>4)
BC-819 + gemcitabine	100%		
Gemcitabine	37%	63%	
No treatment			100%
